# Chronic obstructive pulmonary disease and inhaled steroids alter surfactant protein D (SP-D) levels: a cross-sectional study

**DOI:** 10.1186/1465-9921-9-13

**Published:** 2008-01-28

**Authors:** Michael W Sims, Ruth M Tal-Singer, Sonja Kierstein, Ali I Musani, Michael F Beers, Reynold A Panettieri, Angela Haczku

**Affiliations:** 1 Department of Medicine, University of Pennsylvania, Philadelphia, PA, USA; 2 COPD Biology, GlaxoSmithKline, King of Prussia, PA, USA

## Abstract

**Background:**

Surfactant protein D (SP-D), an innate immune molecule, plays an important protective role during airway inflammation. Deficiency of this molecule induces emphysematous changes in murine lungs, but its significance in human COPD remains unclear.

**Methods:**

We collected bronchoalveolar lavage fluid from 20 subjects with varying degrees of COPD (8 former smokers and 12 current smokers) and 15 asymptomatic healthy control subjects (5 never smokers, 3 remote former smokers, and 7 current smokers). All subjects underwent a complete medical history and pulmonary function testing. SP-D was measured by Enzyme-Linked ImmunoSorbent Assay. Statistical analysis was performed using nonparametric methods and multivariable linear regression for control of confounding. The effect of corticosteroid treatment on SP-D synthesis was studied *in vitro *using an established model of isolated type II alveolar epithelial cell culture.

**Results:**

Among former smokers, those with COPD had significantly lower SP-D levels than healthy subjects (median 502 and 1067 ng/mL, respectively, p = 0.01). In a multivariable linear regression model controlling for age, sex, race, and pack-years of tobacco, COPD was independently associated with lower SP-D levels (model coefficient -539, p = 0.04) and inhaled corticosteroid use was independently associated with higher SP-D levels (398, p = 0.046). To support the hypothesis that corticosteroids increase SP-D production we used type II alveolar epithelial cells isolated from adult rat lungs. These cells responded to dexamethasone treatment by a significant increase of SP-D mRNA (p = 0.041) and protein (p = 0.037) production after 4 days of culture.

**Conclusion:**

Among former smokers, COPD is associated with lower levels of SP-D and inhaled corticosteroid use is associated with higher levels of SP-D in the lung. Dexamethasone induced SP-D mRNA and protein expression in isolated epithelial cells *in vitro*. Given the importance of this molecule as a modulator of innate immunity and inflammation in the lung, low levels may play a role in the pathogenesis and/or progression of COPD. Further, we speculate that inhaled steroids may induce SP-D expression and that this mechanism may contribute to their beneficial effects in COPD. Larger, prospective studies are warranted to further elucidate the role of surfactant protein D in modulating pulmonary inflammation and COPD pathogenesis.

## Background

Chronic obstructive pulmonary disease (COPD) is characterized, in part, by an abnormal inflammatory response of the lung to noxious particles or gases [1], chiefly cigarette smoke. Innate immunity is the vanguard of this multifactorial inflammatory response to cigarette smoke-induced lung injury and may play an important role in COPD pathogenesis. Despite a wealth of evidence suggesting that surfactant protein D (SP-D) modulates innate immunity in the lung, little is known about its role in human COPD.

Surfactant protein D (SP-D), together with surfactant protein A and mannose binding lectin, is a member of the innate immune "collectin" family of structurally related Ca^2+ ^dependent lectins that share ***col ***lagen-like N-terminal tails and globular ***lectin ***heads containing C-type carbohydrate recognition domains. Produced in alveolar type-II cells and Clara cells, SP-D is a 43-kD monomer that forms a higher order quaternary structure (usually a dodecamer assembled from 4 homotrimers). SP-D binds to and enhances clearance of a wide variety of pathogens [2-9], promotes phagocytosis of apoptotic cells [10,11] and inhibits pro-inflammatory cytokine release by effector cells [6,12-14]. SP-D deficient mice display an abnormal pulmonary phenotype characterized by activated alveolar macrophages, increased levels of matrix metalloproteases and emphysematous changes in the lung parenchyma [15-17]. We and others have previously demonstrated that these mice are more susceptible to lung injury from a variety of insults including bleomycin, ozone challenge, allergic sensitization and viral, bacterial or pneumocystis infection [13,14,16-20]. Because of the immunoprotective properties of SP-D, constitutive expression in the proximal and distal airspaces appears essential in order to maintain an immunologically hyporeactive tissue milieu under normal (non-infectious) conditions.

The mechanisms that regulate expression and function of this immunoprotective molecule are unknown. Previous studies have found decreased levels of SP-D in the lung [21,22] in association with cigarette smoking, but these studies have not controlled for the potential confounding effects of COPD. We hypothesized that chronic cigarette smoking and COPD would each be independently associated with lower SP-D levels in the lung. In order to determine the association between pulmonary SP-D levels, cigarette smoke exposure, and COPD, we conducted a cross-sectional study of healthy nonsmokers, healthy smokers, and current or former smokers with varying degrees of COPD.

## Methods

### Human subjects

To study the role of SP-D in COPD, we recruited 20 subjects with varying degrees of COPD (8 former smokers and 12 current smokers) and 15 asymptomatic healthy control subjects (5 never smokers, 3 remote former smokers, and 7 current smokers), utilizing direct advertising in the Philadelphia area. Volunteers deemed eligible after a preliminary phone screen were scheduled for a screening visit, during which a detailed medical history, tobacco history, medication history, and physical examination were performed. Smoking status was confirmed by urine cotinine levels in all subjects. All subjects underwent spirometry, lung volume assessment by plethysmography, and measurement of the diffusing capacity for carbon monoxide. To qualify for the healthy non-smoker cohort, subjects were required to be asymptomatic non-smokers with a lifetime tobacco exposure of <10 pack years and no tobacco in the last year. In addition, they were required to demonstrate normal pulmonary function at screening. Healthy smokers were required to be current, asymptomatic smokers with a lifetime tobacco exposure of >10 pack years and normal pulmonary function at screening. COPD subjects were required to have a lifetime tobacco exposure of >10 pack years and to demonstrate incompletely reversible airflow obstruction with a post-bronchodilator forced expiratory volume in one second (FEV_1_) <80% predicted on screening spirometry. Disease severity was established using the Global Initiative for Chronic Obstructive Lung Disease (GOLD) classification [1]. Exclusion criteria included a history of asthma or alpha-1 antitrypsin deficiency, significant medical disease other than COPD (benign conditions such as well-controlled hypertension on minimal medication were allowed), infection in the 3 weeks prior to screening, hospital admission in the 3 months prior to screening, and prior lung resection. Informed consent was obtained from all subjects and the study protocol was approved by the Institutional Review Board of the University of Pennsylvania.

All included subjects underwent bronchoscopy one or more times during the study. At each bronchoscopy, bronchoalveolar lavage fluid (BALF) was collected by sequential instillation of three 50 mL aliquots of sterile saline into a subsegmental bronchus followed by gentle syringe suction. BALF was centrifuged at 1100 rpm for 10 minutes and the cell-free supernatant was separated from the cell pellet and preserved with a protease inhibitor tablet (Roche Diagnostics, from Fisher Scientific, NC9225286) before freezing for subsequent batched analysis.

Of the 35 subjects included in this study, 9 underwent only one bronchoscopy, 22 underwent 2 separate bronchoscopies (each with a separate BALF sample), and 4 underwent 3 bronchoscopies, depending on their participation in prior observational bronchoscopy studies at our institution. Bronchoscopies for any given subject were separated by a minimum of 3 months and no more than 9 months. For each subject, the average of all SP-D levels obtained from the multiple BALF samples was used for all analyses.

### Human SP-D ELISA

Levels of SP-D and Clara cell protein (CCP-16, an anti-inflammatory protein secreted by Clara cells) were measured in the cell free supernantant of BALF using an Enzyme-Linked ImmunoSorbent Assay (ELISA) kit (Biovendor Inc, Brno, Czech Republic). ELISA was performed according to the manufacturer's instructions. Total protein was assessed by standard Bradford assay. Given that the ELISA measures absolute SP-D or CCP-16 concentrations we are presenting the levels without normalization for total protein content of the BAL. SP-D levels normalized to the optical density obtained for protein measurements did not significantly alter the results (data not shown).

### Alveolar Type II Cell Culture

Cells were isolated from adult rat lungs digested by collagenase I and collagenase IA (Sigma, St. Louis, MO) as previously described ([13,23,24]). Briefly, non adherent cells were plated in 35 mm plastic culture dishes (2–2.5 × 10^6 ^cells/ml) in Waymouth's MB 752/1 Medium (Invitrogen, Carlsbad, CA) with 10% fetal bovine serum and incubated at 37°C, 5% CO_2 _overnight (day 0). (Approximately 40–60% of all the cells plated were type II cells. This proportion remained constant throughout the 4-day culture period.) 24 hours later (day 1) cells were washed and further incubated in serum-free Waymouth's medium with 8-Br-cAMP (100 μM) and isobutylmethylxanthine (100 μM) with or without dexamethasone (10 nM) from Sigma Chemical Co. (St. Louis, MO)] for an additional 3 days. Cells were re-fed with fresh medium on day 3 and were harvested on day 0, and day 4. Three individual experiments were performed, each using duplicate cultures.

### Western Blot and Northern Blot Analysis

To detect intracellular surfactant proteins, the cell pellet was sonicated and the supernatant was concentrated by evaporation. Western blot analysis was performed as previously described [25]. Membranes were incubated with rabbit polyclonal anti-SP-D (1:10,000) or with rabbit polyclonal anti-SP-A (1:7,500).

The 386-nucleotide rat cDNA probe for SP-D was newly prepared using Reverse Transcription-Polymerase Chain Reaction (RT-PCR) and the Retroscript Kit (Ambion, Austin, TX). Total RNA was isolated from type II cells by RNeasy kit (Qiagen, Valencia, CA) and Northern blot analysis was performed as previously described [25].

### Statistical analysis

In univariate analysis, SP-D levels were tested for association with age, sex, race, disease status, smoking status, total smoke exposure in pack-years, years since quit smoking, and inhaled corticosteroid use. The Wilcoxon's ranksum test or Kruskal-Wallis test were used for categorical variables with two or more than two levels, respectively, and the Spearman test for correlation was used for continuous variables. The association between disease status and SP-D level in BALF was stratified by smoking status to control for possible confounding. Among former smokers, SP-D levels approximated a normal distribution; as a result, multivariable linear regression was used to test whether the association between disease status and SP-D levels within this group was independent of the effect of other measured variables. Covariates were considered confounders if they altered the association between disease and SP-D levels by 15% or more.

Further analyses included a non-parametric test for trend [26] for SP-D levels among subjects categorized by the combination of smoking and disease status, and a linear regression for SP-D levels among all subjects with smoking status included as a categorical covariate rather than as a restricting factor. Bootstrap resampling was conducted to assess the stability of our observed associations among 1000 repeated random samples of our dataset, using the "bootstrap" command in STATA. This method creates numerous datasets of equal size by random sampling of observations in the original dataset with replacement and then recursively conducts the specified statistical test on each new random sample. Confidence intervals were then identified by the percentile method (i.e. selecting the 2.5 and 97.5 percentile of the distribution of observed coefficients). Lastly, one-way analysis of variance was used to calculate the within-subject variability in SP-D level for subjects with multiple samples available. All analyses were conducted using STATA v.8 (StataCorp LP, College Station, TX).

## Results

### Patient characteristics

We recruited 20 subjects with varying degrees of COPD (8 former smokers and 12 current smokers) and 15 asymptomatic healthy control subjects (5 never smokers, 3 remote former smokers, and 7 current smokers). Subject characteristics by disease status are listed in Table 1. Among subjects with COPD, 9 were classified by the Global Initiative for Chronic Obstructive Lung Disease (GOLD) criteria as GOLD 2 in severity, 8 were classified as GOLD 3, and 3 were classified as GOLD 4. Although GOLD 4 subjects had lower SP-D levels than GOLD 2 and 3 subjects (median SP-D levels 201 ng/mL for GOLD 4 and 461 ng/mL for GOLD 2 and 3), this difference did not reach statistical significance (p = 0.37). As a result, all GOLD stages were pooled together for further analyses. Healthy control subjects were significantly younger than COPD subjects (median ages 51 and 59, respectively, p = 0.02). Although not statistically significant, there were also differences in both gender and race between healthy controls and COPD subjects. Therefore, we adjusted for each of these differences in the primary analysis. As shown in Table 1, lung function parameters were lower while tobacco exposure was greater among those with COPD relative to healthy controls.

**Table 1:** Subject characteristics by disease status*

	Healthy (n = 15)	COPD (n = 20)	p value
Age, years	51 (47 – 60)	59 (54 – 61)	0.02
Sex, n (%)			
Male:	9 (60%)	8 (40%)	0.24
Female:	6 (40%)	12 (60%)	
Race, n (%)			
African-American:	8 (53%)	6 (30%)	0.12
White:	6 (40%)	14 (70%)	
Asian:	1 (7%)	0	
Smoking status, n (%)			
Never:	5 (33%)	0	0.03
Fomer:	3 (20%)	8 (40%)	
Current:	7 (47%)	12 (60%)	
Tobacco exposure, pack years	12.5 (0 – 30)	53.3 (43 – 74.3)	<0.0001
Inhaled corticosteroid (ICS) use, n (%)	0 (0%)	11 (55%)	0.001
ICS use by smoking status, n (%)			
Never:	0 (0%)	0 (0%)	N/A
Fomer:	0 (0%)	4 (50%)	0.24
Current:	0 (0%)	7 (58%)	0.02
FEV_1_, %predicted	102% (96% – 109%)	48% (34% – 65%)	<0.0001
FVC, %predicted	100% (88% – 108%)	80% (68% – 94%)	0.001
FEV_1_/FVC, %predicted	100% (98% – 106%)	59.5% (48.5% – 74.5%)	<0.0001
TLC, %predicted	105% (90% – 111%)	117% (106% – 127%)	0.007
RV, %predicted	98% (88% – 109%)	165.5% (145% – 199.5%)	<0.0001
DLCO, % predicted	99% (84% – 117%)	61.5% (48.5% – 68%)	<0.0001
SP-D, ng/mL	714 (445 – 1078)	449 (310 – 664)	0.03
SP-D by smoking status, ng/mL			
Never:	1078 (1065 – 1239)	N/A	N/A
Fomer:	1067 (861 – 1110)	502 (449 – 765)	0.01
Current:	445 (334 – 704)	334 (290 – 533)	0.45

*All values expressed as median (IQR) unless otherwise specified

Previously published studies on BALF SP-D levels from a wide range of laboratories have shown only single sample measurements. To investigate the variability in individual BALF SP-D levels over time, we compared SP-D levels in repeated BALF samples from subjects who underwent multiple bronchoscopies as described in the Methods. One-way analysis of variance revealed marked within-subject variability over time (standard deviation 215 ng/ml) in BALF SP-D levels from repeat samples obtained within 3–9 months. This value was nevertheless lower than the between-subject variability (standard deviation 306 ng/ml). To attain the most representative measure for further analyses we used the average of SP-D levels obtained from each subject.

### Cigarette smoking was associated with reduced SP-D levels

Smoking status had a substantial impact on SP-D levels, with former smokers having levels intermediate between never smokers and current smokers (median SP-D levels 1078 ng/mL, 724 ng/mL, and 363 ng/mL for never, former, and current smokers, respectively, p = 0.04 for the difference between former and never smokers, p = 0.004 for the difference between current and never smokers). Importantly, all former smokers in this cohort had quit a minimum of 1.5 years prior to bronchoscopy, eliminating the possibility that the lower levels in former smokers were due to bias from recent cessation.

### COPD was independently associated with reduced SP-D levels

**Figure 1:**
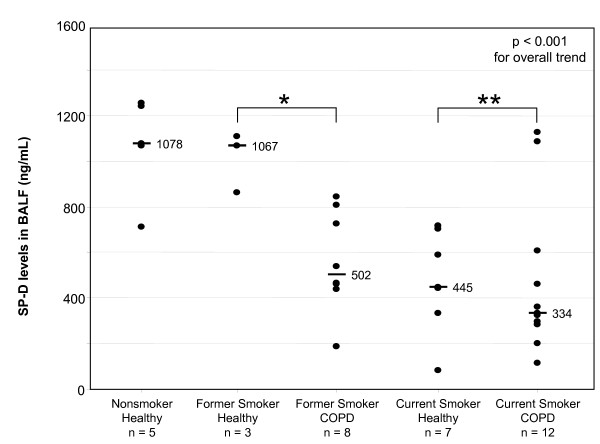
**SP-D levels in BALF according to smoking and disease status**. P value shown on the graph is for the overall trend in SP-D levels by a nonparametric test for trend. * p = 0.01 for the difference between healthy former smokers and former smokers with COPD. ** p = 0.45 for the difference between healthy current smokers and current smokers with COPD.

Overall, subjects with COPD had lower SP-D levels in their BALF compared with healthy subjects (median SP-D levels 449 ng/mL and 714 ng/mL, respectively, p = 0.03). In order to determine if the effect of disease status was independent of the effect of smoking status, we stratified subjects by smoking status and re-examined the effect of disease status among former smokers and current smokers, separately. Among current smokers, those with COPD had only marginally lower SP-D levels in their BALF relative to healthy smoking controls, but this difference was not statistically significant (p = 0.45). However, among former smokers, COPD subjects had significantly lower SP-D levels in their BALF relative to healthy controls (p = 0.01), suggesting that COPD is associated with lower levels of SP-D in the lung independent of smoking status (Figure 1).

### Inhaled corticosteroid use was independently associated with SP-D levels

Since previous studies from our laboratory suggested that corticosteroids may increase SP-D expression [13,24], we examined inhaled corticosteroid (ICS) use as a potential confounder in our study. In univariate analysis, among former smokers with COPD, ICS use was associated with a trend toward higher SP-D levels in comparison to those not using ICS (median SP-D levels 765 ng/mL and 463 ng/mL, respectively, p = 0.15). ICS use was not associated with increased SP-D levels among current smokers with COPD.

Next, we used multivariable linear regression to test whether the association between disease and SP-D levels among former smokers was independent of ICS use. Interestingly, when ICS use was included as a categorical variable in the model with disease status, both maintained an independent association with SP-D levels. Indeed the magnitude of association between disease status and SP-D levels increased. Even when age, sex, race, and tobacco exposure in pack-years were sequentially forced into the model, both disease and ICS use retained independent associations with SP-D levels (Table 2), eliminating the possibility that our observed associations were due to confounding by these factors.

**Table 2:** Multivariable analysis among former smokers

Variables Included	Model Coefficient	p value
B_0_	1342	0.07
COPD (vs. Healthy)	-539	0.04
ICS use	398	0.046
Age	-10	0.34
Sex	110	0.53
Race (African-American vs. White)	216	0.26
Tobacco (pack-years)	2.0	0.52

In order to further examine the stability of our findings, we employed bootstrap resampling, creating 1000 replicates for the model including disease status and ICS use. This analysis confirmed that the associations observed for disease status and use of inhaled corticosteroids were robust (model coefficients [95% CI] were -600 [-826, 0.0] and 290 [13, 516], respectively).

### Effects of dexamethasone on type II alveolar epithelial cell SP-D levels

**Figure 2:**
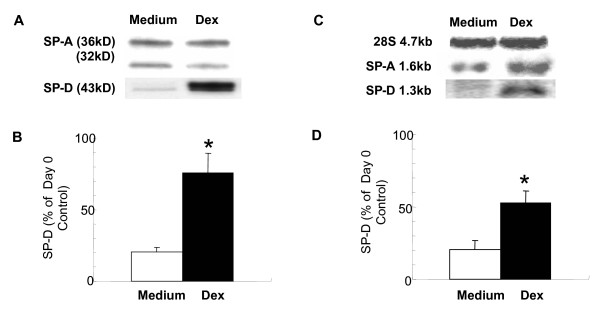
**Dexamethasone upregulates SP-D but not SP-A production by type II cells *in vitro***. Cells were isolated from lungs of adult rats and cultured in Waymouth's medium with 10% FBS, with 8-Br-cAMP (100 μM) and isobutylmethylxanthine (100 μM) with or without dexamethasone (10 nM). Cell lysates were prepared from freshly isolated cells and at the end of a 4-day culture period (A): SP-A and SP-D production was analyzed by Western blot. (B): Results are expressed as % of the "day 0" value. *p = 0.037 (n = 3) (C): Northern blot analysis of SP-D and SP-A mRNA expression was performed on total RNA (10 μg) extracted from type II cells as described. SP-D and SP-A cDNA probes labeled with [α^32^P] dCTP were hybridized to mRNAs on nitrocellulose membranes. (D): Intensity was quantified by densitometric scanning and the hybridization signals were normalized to 28S rRNA probe labeled with [γ^32^P] ATP. mRNA content is expressed as % of the "day 0" value. *p < 0.041 Mean ± SEM was calculated after deriving the average of the results from three independent experiments.

To investigate whether corticosteroids would be capable of directly affecting SP-D synthesis we isolated type II alveolar epithelial cells from adult rat lungs. Cells were cultured for 4 days in the presence or absence of dexamethasone (10 ng/ml). Figure 2 demonstrates that dexamethasone induced a significant increase in SP-D expression (p = 0.037). This increase was commensurate with a raise in SP-D mRNA in these cells (p = 0.041). The effects of dexamethasone were specific because protein expression of the other lung collectin, SP-A did not increase in the presence of this glucocorticoid. These data suggest that dexamethasone is capable of directly enhancing SP-D production by type II alveolar epithelial cells.

### COPD selectively affected SP-D levels in the BALF

To exclude the possibility that lower levels of SP-D among former smokers with COPD were due to a non-specific loss of pulmonary proteins through epithelial destruction, we re-examined our multivariable model for former smokers using SP-D levels normalized to total protein concentration. This model yielded comparable results for the effect of disease on SP-D levels (model coefficient [95% CI] -1162 [-2078, -257], p = 0.02) even after controlling for age, race, pack-years of tobacco use, and ICS use (only when sex was included in the model did the coefficient rise to -981 with a p value of 0.079). In addition, we analyzed Clara cell protein (CCP-16, a protein produced by Clara cells) in the BALF in comparison with SP-D levels. Although former smokers with COPD did have a small but statistically significant reduction in CCP-16 levels compared to healthy former smokers (median CCP-16 levels 9.2 mcg/mL vs. 22.6 mcg/mL, respectively, p = 0.04), this difference disappeared with control for any potential confounders, including age, race, sex, pack-years of tobacco use, or ICS use. This sharply contrasted with the effect of disease on levels of SP-D, which remained statistically significant despite inclusion of all of these confounders, simultaneously. Thus, the reduced SP-D levels in association with COPD appear to be a specific phenomenon rather than simply reflecting an overall decrease in pulmonary proteins due to loss of airway epithelium.

To further investigate the relationship between smoking and COPD with regard to SP-D levels, we subdivided our cohort into 5 groups consisting of healthy never smokers (n = 5), healthy former smokers (n = 3), former smokers with COPD (n = 8), healthy current smokers (n = 7), and current smokers with COPD (n = 12). We then analyzed pulmonary SP-D levels by this categorization and found that there was a very strong pattern of progressive decline in SP-D levels as determined by a non-parametric test for trend (p < 0.001; Figure 1). These data support our observation that both smoking status and COPD have independent effects on SP-D levels. This analysis also highlighted the effect of smoking among healthy subjects: healthy current smokers had lower levels of SP-D in the BALF relative to healthy never smokers (median SP-D levels 445 ng/mL and 1078 ng/mL, respectively, p = 0.007) but there was no difference in SP-D levels between healthy former smokers and healthy never smokers (median SP-D levels 1067 ng/mL and 1078 ng/mL, respectively, p = 0.65).

In order to determine if the association between COPD and SP-D levels would remain despite including all smoking strata, we also repeated our multivariable linear regression on the entire cohort (Table 3), including smoking status as a categorical covariate. Due to probable collinearity, we were unable to develop a stable model including an interaction term between disease status and smoking status. When both variables were included in the model as main effects without an interaction, the association of smoking status with SP-D levels was stronger than that of disease status with SP-D levels. When race was added to the model with smoking status (omitting disease status), the estimates of association for smoking strata were not altered. However, African-American race was associated with significantly higher SP-D levels compared to white race.

**Table 3:** Multivariable analysis among all subjects

Variables Included	Model Coefficient	p value
B_0_		
Smoking status (former vs. never)	970	< 0.001
(current vs. never)	-356	0.02
	-628	< 0.001
Race (African-American vs. White)	248	0.01
(Asian vs. White)	103	0.71

## Discussion

The relationship between smoking and innate lung immunity remains poorly defined. In this study, we demonstrate a significant relationship between COPD and lower levels of pulmonary SP-D that is independent of current cigarette smoke exposure and controlled for multiple potential confounders. In addition, for the first time, controlled data indicates an association between the use of inhaled corticosteroids and increased levels of pulmonary SP-D. Given the importance of SP-D as a regulator of innate immunity and inflammation in the lung [13-15], we speculate that depletion and/or dysfunction of this protective lung collectin may contribute to disease pathogenesis and progression. Our intriguing observations among former smokers suggest that SP-D expression may be a novel biomarker reflecting the presence of disease and steroid effects, and may also suggest a potential role for this molecule in COPD pathogenesis.

The association between COPD and SP-D levels was not statistically significant among current smokers or when smoking status was included as a categorical covariate for the entire cohort. We postulate that the acute effect of current cigarette smoke exposure on pulmonary SP-D levels overshadows the chronic effect of COPD, such that the effect of disease was only detectable among former smokers. Further studies with larger numbers of patients, and thus greater statistical power, will be required to determine whether an effect of disease can be detected among current smokers.

Similarly, subjects in our study with very severe COPD (GOLD 4) had lower levels of SP-D compared with subjects with GOLD stage 2 or 3 disease, but this difference was not statistically significant. While we hypothesize that greater disease severity is associated with lower SP-D levels, our study was underpowered to detect this difference. Clearly, further studies with larger numbers of subjects will be required to ultimately prove or disprove this hypothesis.

In our study, no distinction was made between COPD subjects fitting the emphysema phenotype and those fitting a more chronic bronchitic phenotype. Although SP-D may be differentially affected in these disparate phenotypes, any such difference would likely bias our study toward the null hypothesis; that is, such differences would increase the overall variability in SP-D levels among COPD subjects and thus increase the likelihood of failing to detect an association. Thus, that this variability would cause a spurious difference (i.e. a type-I error) is unlikely. Similarly, although definitive exclusion of asthma in clinical studies of COPD subjects with symptomatic airflow obstruction is quite challenging, the likelihood of asthma confounding our results is low. We carefully excluded subjects with a history of asthma, our COPD subjects were relatively old (median age 59, minimum age 49) with a substantial tobacco exposure history (median 53 pack-years, minimum 23 pack-years) and incompletely reversible airflow obstruction.

It is possible that SP-D levels are lower in those with disease as a result of disease-related epithelial destruction that depletes the Clara cells and Type-II alveolar cells responsible for SP-D production. As such, SP-D could simply be acting as a marker of parenchymal destruction in COPD. Arguing against this is the fact that normalizing SP-D levels to the optical density for protein measurement did not substantially change the results of our multivariable model and that CCP-16 levels did not appear to be altered by disease independent of confounding. These results indicate that the ability to produce SP-D is unlikely to be a simple reflection of the extent of emphysematous changes in the distal air spaces.

Regardless of whether lower SP-D expression in COPD subjects is due to parenchymal destruction, depletion of this protective molecule may still have a substantial effect on subsequent disease progression and susceptibility to infection. In murine models, mice deficient in SP-D display a markedly abnormal phenotype with pulmonary emphysema, accumulations of activated macrophages, and increased levels of matrix metalloproteases [15,20,27-29], all of which are characteristic of emphysema in humans. These mice are more susceptible to lung injury from a variety of insults including bleomycin, ozone challenge, allergic sensitization and viral, bacterial or pneumocystis infection [13,14,16-20]. Further, treatment of SP-D deficient mice with a recombinant fragment of SP-D has been demonstrated to reverse these pathologic changes [10,30], suggesting that SP-D is more than just a marker of parenchymal destruction and appears to play an important regulatory role in the lung. These data suggest that SP-D modulates the immune response to pulmonary infection and the inflammatory response to lung injury. Thus, to the extent that these murine models are reflective of human disease, it appears that relative deficiency of this protective molecule may confer increased susceptibility to infection and disease progression.

Interestingly, in our model including all 35 subjects, African-American race was associated with higher SP-D levels independent of smoking status. While possible explanations such as differences in types of tobacco smoked or other smoking-related behaviors are plausible, this effect could potentially represent genetically regulated differences in expression. Indeed, in humans allelic variations have been shown to be strongly associated with differences in SP-D serum levels [31]. Heidinger et al. reported that a single SP-D gene haplotype had a highly significant negative association with serum SP-D levels. In addition, several single nucleotide polymorphisms in genes encoding surfactant proteins have been linked to COPD in a Mexican population [32]. Whether genetic predisposition to low levels of SP-D production would increase susceptibility to develop COPD upon exposure to cigarette smoke, however, needs further clarification. Importantly, our observed association between COPD and lower SP-D levels among former smokers remained significant despite controlling for race in our multivariable analysis, excluding the possibility that racial differences in SP-D expression confounded our results.

Our multivariate analysis suggested that ICS use was independently associated with higher SP-D levels in the lung of former smokers with COPD. Corticosteroids have been shown to induce expression of SP-D in the fetal lung tissue of both rats [24,33-35] and humans [36-38]. Here we investigated adult type II alveolar epithelial cells isolated from rat lungs. We found that similarly to fetal cells, SP-D synthesis depended on constitutive presence of dexamethasone. Indeed this glucocorticoid significantly enhanced expression of both mRNA and protein for SP-D. Given that there are several high potency inhaled steroids widely used to treat COPD and that these steroids are deposited directly on the epithelium, the site of SP-D production, it is reasonable to suspect that these medications may have effects on local SP-D gene expression. Given the high level of evolutional conservation of the SP-D gene promoter sequence between rats and humans, we speculate that inhaled corticosteroids induce expression of SP-D in the adult human lung. Further, this mechanism may enhance the anti-inflammatory effects of inhaled corticosteroids in COPD.

In summary, we have demonstrated for the first time an association between decreased SP-D levels in the lung and COPD that is independent of current cigarette smoke exposure. We hypothesize that chronic exposure to cigarette smoke reduces SP-D expression and that subsequent relative deficiency of this protective molecule impairs innate immunity and contributes to the pulmonary inflammation characteristic of COPD. In this cross-sectional study, we were unable to determine the temporal association between lower SP-D levels and development of disease, making causal inference tentative at best. Larger studies with prospective follow-up are warranted to determine whether our observations are reproducible and predictive of subsequent clinical outcomes. Our study emphasizes that the relationship between COPD, corticosteroid therapy, and the structure and function of pulmonary SP-D merits further investigation.

## Conclusion

Among former smokers, COPD is associated with lower levels of SP-D and inhaled corticosteroid use is associated with higher levels of SP-D in the lung. Given the importance of this molecule as a modulator of innate immunity and inflammation in the lung, low levels may play a role in the pathogenesis and/or progression of COPD. We speculate that inhaled steroids may induce SP-D expression and that this mechanism could contribute to their anti-inflammatory effects in COPD. Larger, prospective studies are needed to further elucidate the role of surfactant protein D in modulating pulmonary inflammation and COPD pathogenesis.

## Abbreviations

BALF: bronchoalveolar lavage fluid; CCP-16: Clara cell protein 16; COPD: chronic obstructive pulmonary disease; ELISA: enzyme-linked immunosorbent assay; FEV_1_: forced expiratory volume in one second; FVC: forced vital capacity; FEV_1_/FVC: ratio of forced expiratory volume in one second to forced vital capacity; GOLD: Global Initiative for Chronic Obstructive Lung Disease; ICS: inhaled corticosteroid; RV: residual volume; SP-D: surfactant protein D; TLC: total lung capacity

## Competing interests

RTS is a full-time employee of GlaxoSmithKline. MS, AH, and RAP receive funds from GlaxoSmithKline in the form of research grants.

## Authors' contributions

Michael Sims managed recruitment and clinical characterization of subjects, conducted all statistical analyses, and wrote the manuscript. Ruth Tal-Singer helped conceive the study and provided substantive feedback on the manuscript. Sonja Kierstein conducted all laboratory assays. Ali Musani performed all bronchoscopies and collected the BALF samples. Michael Beers assisted with data interpretation and editing of the manuscript. Rey Panettieri and Angela Haczku contributed equally to the conception of the study and editing of the manuscript. All authors read and approved the final manuscript.
